# Some Prejudices

**Published:** 1914-09

**Authors:** 


### Some Prejudices

Medical writers persist in referring to **moving picture** shows as filthy, badly ventilated places of assignation, where immoral pictures are shown and eye-strain produced, and where poor people waste their money. We can name seven picture show houses in Buffalo which, for cleanliness and architectural planning for sanitary cleaning, ventilation, seating comfort, provision against fire or panic surpass any of the regular theaters and most churches, school houses and other places of public assembly. We do not doubt but that improper acquaintances are made through the medium of the picture show.

So they are through any other meeting place, not excluding the church. Many films portray silly subjects and some give provokingly incorrect views of various topics. So do plays, books, magazine articles, etc. In a very moderate observation but including various cities of this country and Europe, we do not remember ever to have seen a film to which exception could be taken on moral grounds, barring reproductions of ballets, etc., of precisely the same nature as shown on the regular stage. We have heard of one or two "problem plays" to which some persons objected seriously; one of these was given under the auspices of a woman's benevolent society. The status of such plays is sub judice. As to extravagance, we are inclined to think that some persons will spend too much money on pleasure anyway. One of our business acquaintances confesses to being a picture show fiend, attending one every afternoon and evening if not prevented. But even such excess represents only about the price of a single admission to a regular theatre and no more waste of time than most men indulge in for various kinds of amusement. That poor people can have an evening's recreation, at an early hour and for a nickel seems to us to be a distinct advantage, even if it involves the same aggregation of exhalations and potential risk of infection, to be encountered wherever they are gathered together. And, in the long run, those who attend the "movies" pick up a surprising amount of information.

Medical as well as other periodicals have joined in the attack on the **Tango** and, right in our own territory, teachers have been required to sign an agreement not to dance it, before being awarded contracts. We have no intention of discussing dancing in general, from either the hygienic or moral standpoint, or either to defend or to condemn. But, why should the tango be singled out for special invective? Two or three years ago, what was called the tango was a decidedly sudorific and somewhat wiggly dance, ungraceful unless done by experts and so rapid that extemporaneous leading was impossible. Except that, deliberately or unconsciously, it might call the gluteal muscles into play, it could scarcely be considered objectionable in the usual sense of the word. Last year, it was superceded by the so-called Argentine tango which we understand bears the same relation to the original, ancient South American national dance, that a quiz compend on anatomy bears to Gray's work. This tango, so far as we have seen it, is slow in both the literal and the slang sense, and is objectionable only because it requires a traffic squad to render it possible, diverts the attention of the dancers from the rhythm and music to counting and leads to altercations between otherwise peaceable persons if there is a disagreement as to the count, or if a wall or another couple intervenes at the wrong place. As an exhibition dance, with many complicated figures, the tango is beautiful to witness.

The **cigarette** is coming in for concentrated attack by many business firms. As in the last case, we have no defense for smoking in general but wish simply to call attention to the fact that a cigarette smoked as far as possible without a special holder, weighs about 3/4 of a gram, while a cigar similarly smoked, weighs about 9 grams. It has been shown by medical investigation, in this country and Great Britain, that no standard brand of cigarettes contains narcotics—aside from tobacco itself—and that the almost infinitesimal amount of rice paper does not contain arsenic or produce any unusual harmful substance by combustion. It has likewise been shown that, while nicotine exists in tobacco to a dangerous amount, it enters into the smoke only in minute amount, the principal dangers being from carbon monoxid, possibly certain nitrogenous compounds and from acrylic radicles if glycerin has been employed to maintain moisture. It is obvious that, with the exception of the last, the dangers from these products are far less in the combustion of the small, hot spark of the cigarette, freely exposed to oxygen of the air, than from the more sheltered smouldering fire of the cigar or pipe.

